# Four-year follow-up on fatigue and sleep quality of a three-armed partly randomized controlled study in breast cancer survivors with cancer-related fatigue

**DOI:** 10.1038/s41598-022-25322-y

**Published:** 2023-02-15

**Authors:** M. Kröz, F. Quittel, M. Reif, R. Zerm, D. Pranga, C. Bartsch, B. Brinkhaus, A. Büssing, C. Gutenbrunner, the CRF study group, Fadime ten Brink, Bettina Berger, Annette Weninger, Matthias Girke, Augustina Müller-Glinz, Christian Heckmann

**Affiliations:** 1 Research Institute Havelhöhe (FIH), Kladower Damm 221, 14089 Berlin, Germany; 2 Department of Research and Somnology, Arlesheim Hospital, Arlesheim, Switzerland; 3 Institute for Integrative Medicine, University of Witten/Herdecke, Witten, Herdecke, Germany; 4 Society for Clinical Research, Berlin, Germany; 5 Department of Internal Medicine, Havelhöhe Hospital, Berlin, Germany; 6 Center for Research in Medical and Natural Sciences, University of Tübingen, Tübingen, Germany; 7 Charité – Universitätsmedizin Berlin, corporate member of Freie Universität Berlin, Humboldt-Universität zu Berlin, and Berlin Institute of Health, Institute for Social Medicine, Epidemiology and Health Economics, Berlin, Germany; 8 Clinic for Rehabilitative Medicine, Hannover Medical School, Hannover, Germany; 9 University of Witten/Herdecke, Witten, Herdecke, Germany

**Keywords:** Breast cancer, Patient education, Rehabilitation, Quality of life

## Abstract

Cancer-related fatigue (CRF) is a frequent long-term symptom in non-metastasized breast cancer patients (BC). This 4-year follow-up intended to compare the long-term effects of a 10-week multimodal therapy (MT: sleep education, psychoeducation, eurythmy- and painting therapy) and combination therapy [CT: MT plus aerobic training (AT)] to AT-control. BC-patients were randomized or allocated by preference to three arms in a comprehensive cohort study. Primary outcome was a composite score including Pittsburgh Sleep Quality Index (PSQI) and Cancer Fatigue Scale (CFS-D), captured at baseline, after 10 weeks of intervention (T1), 6 months later (T2), and after 4 years (T3). We exploratively tested for superiority of MT and CT versus AT after 4 years (T3) based on the statistical model of the main analysis. Of 126 (65 randomized) BC-patients included, 105 started treatments and 79 were re-assessed for long-term effects (T3). MT and CT were superior over AT after 4 years regarding PSQI/CFS-D and PSQI sum-score, respectively (all *p* < 0.05), but not for CFS-D. The multimodal and combination treatment for breast cancer patients with CRF indicates sustainable long-term superiority over aerobic training for the outcomes sleep quality and combined sleep quality/fatigue. A confirmative randomized controlled trial is warranted.

## Introduction

With the growing amount of therapy options and a growing variety of toxicity and symptom burden in patients with different cancers on the one hand^1^ and improved survival rates for different cancers, including breast cancer, on the other hand^2^, patients’ perspectives, including patient-reported outcomes, have become increasingly important^3^. Regarding breast cancer patients after adjuvant therapies, there are a number of different disabling consequences of breast cancer and breast cancer treatment. Following breast cancer surgery and axillary lymph node dissection lymphedema, shoulder dysfunction, pain and postoperative infection, axillary web syndrome characterized by cordings or musculoskeletal symptoms induced by Aromatase inhibitors can occur which can be treated with tailored oncological rehabilitation concepts^4,5^.

In a British study, 58% of out-patient cancer patients reported that fatigue had affected them in the past months, and more than half of these patients reported that the symptoms were well managed or controlled^6^. Cancer-related fatigue (CRF) is defined “as a distressing, persistent, subjective sense of physical, emotional, and/or cognitive tiredness or exhaustion related to cancer or cancer treatment that is not proportional to recent activity and interferes with usual functioning”^7^.

Out of cancer patients undergoing chemotherapy, 58–94% reported fatigue^7,8^, and out of those with metastasized conditions more than 75% had a symptom burden of fatigue^7^. After 1–5 years after the end of adjuvant therapies, fatigue is an important issue in 28–38% of breast cancer survivors^9–11^ and persists even after 5–10 years, depending on different measures and therapies, in 18–39% of survivors^12–14^. In a long-term follow-up study of 6952 cancer survivors, including 43% of breast cancer patients, the highest symptom scores were measured over 5–16 years with the EORTC QLQ C30 for fatigue and insomnia (between 35 and 40%)^15^. In particular, long-term cognitive fatigue and impairments such as impaired immediate- and delayed verbal memory, processing speed and executive function persist even 20 years after adjuvant chemotherapy^16^. Aerobic training is the therapy with the best available evidence regarding quantity and quality of studies^7,17^ and can be combined in oncological rehabilitation with strength training^5^. Beside aerobic training minor and moderate effect-sizes have also been reported for cognitive behavioral therapy, sleep interventions (education, sleep restriction and stimulus control) and mindfulness-based treatments^7,17^. To improve clinically relevant, sustainable therapy results for breast cancer patients with CRF, we developed a multimodal treatment concept based on a ten-week intervention program integrating psychoeducation (to improve patients’ management competence)^18^, sleep education, sleep restriction, stimulus control (to improve patients’ rest/activity rhythm)^19^, eurythmy therapy^20^ and painting therapy^21^ (to enhance mindfulness-oriented relaxation)^22^. In a pilot study we showed significant improvements in the multimodal treatment group for the Cancer Fatigue Scale (CFS-D) and the Pittsburgh Sleep Quality Index (PSQI)^22^. A second study with a comprehensive cohort design showed significant exploratory superiority in the primary outcome composite (PC-) score of CFS-D/PSQI of the multimodal treatment (MT) group versus aerobic training (AT) after 10 weeks of intervention and of both the multimodal and combination group (CT: MT and AT) 6 months later^23^. The multimodal therapy impacted positively on different health-related quality of life criteria, including less fatigue and fewer insomniac symptoms^24^. In this article we report the long-term effects on the primary outcome—combined PC-score of CFS-D and PSQI—and the secondary outcomes of the single scales CFS-D and PSQI after 4 years and we evaluate if a combination therapy and a multimodal therapy are superior to the control aerobic training only.

## Methods

The prospective open-label, pragmatic three-armed study was designed as a comprehensive cohort study and was conducted in three centers, the Research Institute Havelhöhe and Department of Internal Medicine, Hospital Havelhöhe, Berlin, Center of Integrative Medicine University Witten/Herdecke and Breast Cancer Center, Hospital Herdecke, Herdecke, Department of Rehabilitation Medicine, Hannover Medical School, Hannover, all located in Germany. The trial was conducted from June 2011 to December 2013. Patients were first invited to participate in the study and to be randomized into one of the three treatment arms; if they declined random assignment they could choose their preferred treatment arm. Here, we present a 4-year follow-up undertaken from May 2016 to February 2017. This CRF-2 study is an investigator-initiated trial which was conducted according to the declaration of Helsinki^25^.

### Patients

The patients were recruited through local newspapers, through physicians who contacted their patients, and some patients contacted the study centers on their own accord. After the study was explained to them, each patient signed an informed consent form.

Patients’ eligibility was defined according to the following inclusion criteria:

- Female with breast cancer at the age of 18–75 years, diagnosis of chronic CRF of at least 6 months (Fatigue Numerical Scale (FNS) ≥ 4, Cancer Fatigue Scale (CFS-D) ≥ 24) with a history of a maximum of 36 months since the end of adjuvant therapy (operation, chemo- or radiotherapy) and a maximum of 45 months after the first diagnosis.
- The major exclusion criteria were the following:
  metastases, (radio-) chemotherapy or surgery in the last 6 months, anemia (hemoglobin < 10 mg/dl), other severe chronic conditions (see in detail^23^).

### Treatment allocation and randomization

All patients were informed about the study design and study interventions to support them in their decision to be allocated to one of the intervention groups, based on balanced randomization with a group proportion of 1:1:1 with varying permutation block sizes, or, if they did not wish to be randomized, to choose by their own preference. The randomized allocation was conducted via central randomization at the Institute for Clinical Research (IKF), Berlin.

### Procedures and treatment

Within this trial the following modules were applied in different combinations. The multimodal therapy group received the following therapy modules: psychoeducation, sleep-education, eurythmy therapy and painting therapy. The combination therapy group received the components: psychoeducation, sleep-education, eurythmy therapy, painting therapy and aerobic training. The aerobic training group was applied as a stand-alone therapy (see the therapy components in detail).

### Psychoeducation (in MT/CT)

After a feedback procedure, the psycho-oncologists provided and highlighted information on the understanding of breast cancer and CRF and of dealing with distressing feelings and thoughts, promotion of mental and physical health, social support and communication, personal responsibility, concentration exercises and stress management and aspects of reorientation to improve patients’ self-management capacity to deal with their chronic condition^22,23^. The participants were requested to conduct home-based behavioral exercises^23^.

### Sleep education (in MT/CT)

Patients attended 2 sessions. The first was an information session on the basics of circadian and basic rest/activity rhythms and sleep. This session aimed to contribute to an enhanced understanding of sleep and fundamental biological rhythms, a better ability to improve the adaption of sleep habits and to improve sleep alterations and daytime functioning. Before the intervention started, patients were first asked to fill out a sleep diary. After their first session on sleep hygiene information and recommendation of sleep restriction and stimulus control to improve rest/activity synchronization they were asked again to fill out a second sleep diary to have a basis for further individualized sleep restriction and adaptation at the second session^26–28^ (see^23^).

### Eurythmy therapy (in MT/CT)

Eurythmy therapy is a mindfulness-oriented movement therapy expressing sounds and rhythms as movements and gestures^29^, largely used in both in- and outpatient settings in anthroposophic medicine. To improve cancer-related alterations of rest/activity rhythm, group based exercises were practiced such as I-A-O, clenching–spreading, striding, rhythms/hexameter, the vowel “Ei” and consonants which should achieve a rhythmic stabilization such as L, M and R and O-E-M-L-EI-B-D (the so-called cancer series) was finally introduced and practiced^22,23,29^.

### Painting therapy (in MT/CT)

Painting therapy in the context of our study^30^ aimed to contribute to the regulation of cognitive and affective functions^22^. The therapy sessions started with a 10-min period of drawing shapes. Participants were then invited to paint a development series of paintings from darkness to daylight with a light spectrum, using watercolors and starting with a painting in blue and then progressing step by step to a gradual augmentation of brightness by adding yellow and finally red, resulting in a sunrise^22,23^.

### Aerobic training (in AT/CT)

Aerobic training is in fact the treatment with the best evidence regarding quality and quantity of trials for CRF^17^ and was used as a control arm and implemented in the multimodal-aerobic combination therapy. We wanted to achieve a 70–80% exposure^31^. At baseline, we assessed the patients' performance status with an ergometry test^32^. During the trainer-guided exercise training, performance adjustment was controlled based on heart rate monitor watches^32^. We provided 8 trainer-led 45-min sessions, inclusive of a rest period, and complemented this with home-based training which participants were asked to carry out 3–5 times a week in 30–45 min sessions. Participants documented their training in a protocol (see^23^).

The multimodal treatment was carried out once a week in a 140–165-min session and an additional 15 min for debriefing over ten weeks. According to protocol, the intervention was planned to take a total of 1450 min in multimodal therapy. In the combination group the length of therapy was 165–185 min with an additional 15-min period for debriefing. The intervention was planned to take a total of 1810 min in combination therapy over 10 sessions, once a week. In multimodal therapy and combination therapy, patients were also asked to carry out home-based exercises in eurythmy therapy and psychoeducation and in aerobic training and combination therapy patients were asked to carry out home-based endurance training^23^.

### Outcome measures

The primary outcome in this trial is the composite outcome of the Cancer Fatigue Scale (CFS-D) and the Pittsburgh Sleep Quality Index (PSQI) after 10 weeks of intervention (T1). The results after 10 weeks of intervention (T1: primary outcome) and 6 months later (T2) were published elsewhere^23^. Here we present the follow-up after 4 years.

The therapy appraisal was registered in a five-point Likert scale single item.

The German version of the Cancer Fatigue Scale (CFS-D) is a 15-item scale measuring the construct in a sum scale based on three subscales (physical, cognitive and affective fatigue) with robust reliability and validity^33^. The CFS captures the multidimensionality of fatigue based on a five-point Likert scale with higher values indicating worse fatigue (0–60) and is validated in different languages and was recommended on the NCI website until 2020 as one of ten measures^34–36^.

The Pittsburgh Sleep Quality Index is an international, widely used questionnaire to measure sleep quality^37^ in 19 self-rated items and five external questions. It captures sleep quality in a sum scale on sleep quality score (0–21) with higher scores indicating worse sleep quality based on seven subscales (sleep quality, sleep latency, sleep length, sleep efficiency, sleep disturbances, sleep remedies and daytime fatigue, each 0–3 points) with sufficient reliability and validity^37^.

### Safety

For data on safety see^23^.

### Statistics

#### Testing strategy

To be consistent with the primary analysis of the main study and to avoid an overly multiple testing inflation of the alpha error we first tested the CFS-D and PSQI as a univariate composite score derived from their joint principal component (PC-score)^38^ and both individual questionnaires only subsequently. Yet, since all tests are interpreted in an explorative intention no further adjustments for multiple comparisons were done. Furthermore, since both experimental therapy arms had shown significant improvements versus control six months after the treatment period, the analyses presented here only tested for superiority of multimodal therapy and combination therapy over aerobic training.

#### Sample size estimation

Sample size depended on the number of questionnaires received from the sample of patients included in the main study. Thus, no formal sample size estimation could be done.

#### Statistical analysis

Missing questionnaire items were substituted according to their respective manuals. Due to the long time between primary and 4-year follow-up data collection we refrained from substituting completely missing questionnaires with data of the same or similar patients. Therefore, the analysis was by definition a complete-case analysis.

Statistical analysis primarily followed the approach presented in the primary publication of the study^23^. In brief, a general linear model was used including the baseline score of the analyzed parameter (PC score, CFS-D and PSQI total and sub-scales, respectively) as covariate and the treatment arm and preference/randomization status as independent factors. In addition, the two propensity scores (PS) used in the analysis of the main study were also included as covariates, which aim to account for bias due to treatment allocation by preference versus randomization, and by preferring aerobic training over multimodal therapy, respectively^39^. Differences between treatment arms are shown as mean estimates with 95% confidence intervals. In addition, these differences, and also changes in outcome parameters from baseline within each treatment arm are descriptively expressed as standardized effect sizes to enable a direct comparison of all outcome parameters. All estimates and statistical tests were produced with SAS^®^ version 9.4 (SAS^®^ Institute, Cary, NC, USA, 2016).

### Ethical approval

The study follows the guidelines for clinical trials (Declaration of Helsinki, ICH-GCP). The study was approved by the ethics committee of “Ärztekammer Berlin” (ethics reference number: ETH-06/11, 23.05.2011, with an amendment on 27.04.2015) and confirmed by the ethics committees of the Medizinische Hochschule Hannover (ethics reference number: 1119–2011, 27.06.2011, with an amendment on 23.06.2015) and the University of Witten/Herdecke (ethics reference number: 125/2011, 25.10.2011, with an amendment on 12.11.2015). The study was subjected to GCP-conform monitoring and all included patients signed a written consent. The study is registered in German Clinical Trials Register (DRKS-ID: DRKS00003736; Date of registering 19.06.2012).

## Results

**Figure 1:**
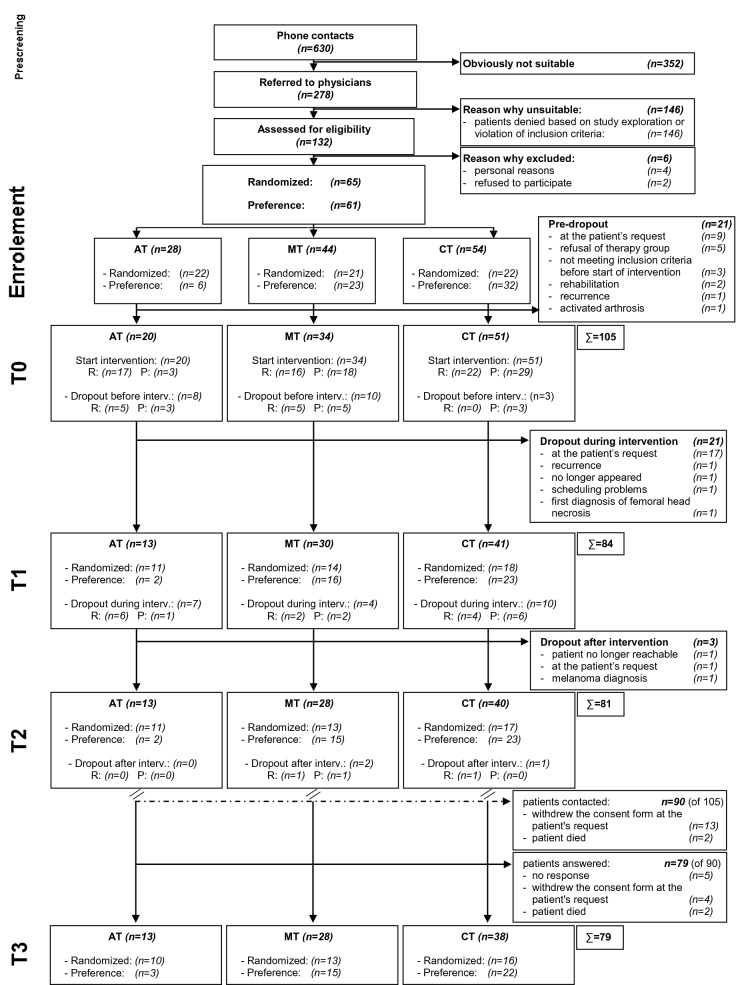
Flowchart of recruitment. *AT* aerobic therapy, *MT* multimodal therapy, *CT* combination therapy, *R* randomized, *P* preference, T0: baseline, T1: after 10 weeks of intervention, T2: after 6 months, T3: after 4 years.

**Figure 2:**
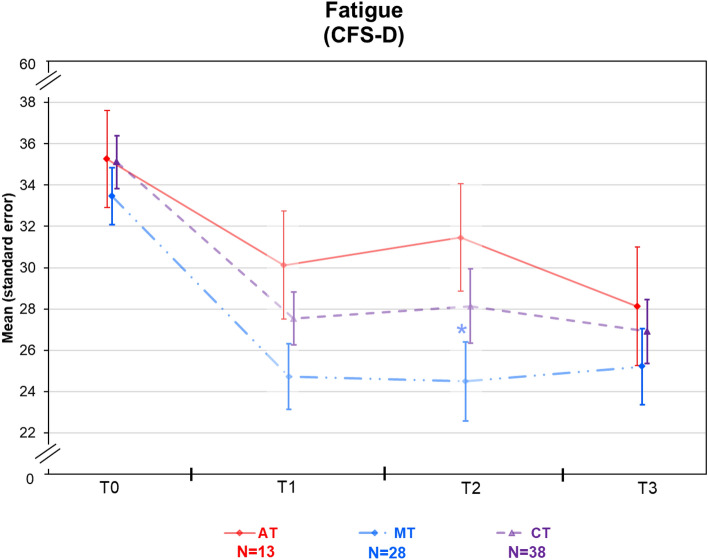
Course of the CFS-D for the 3 study groups (aerobic training: red; multimodal therapy: blue; combination therapy: violet) over 4 years (n = 79), including after 10 weeks of intervention (T1) and 6 months later (T2) in frail color (n = 79). Colored asterisk indicates significant less fatigue in MT versus AT. *CFS-D* cancer fatigue scale, German version, *AT* aerobic therapy, *MT* multimodal therapy, *CT* combination therapy.

**Figure 3:**
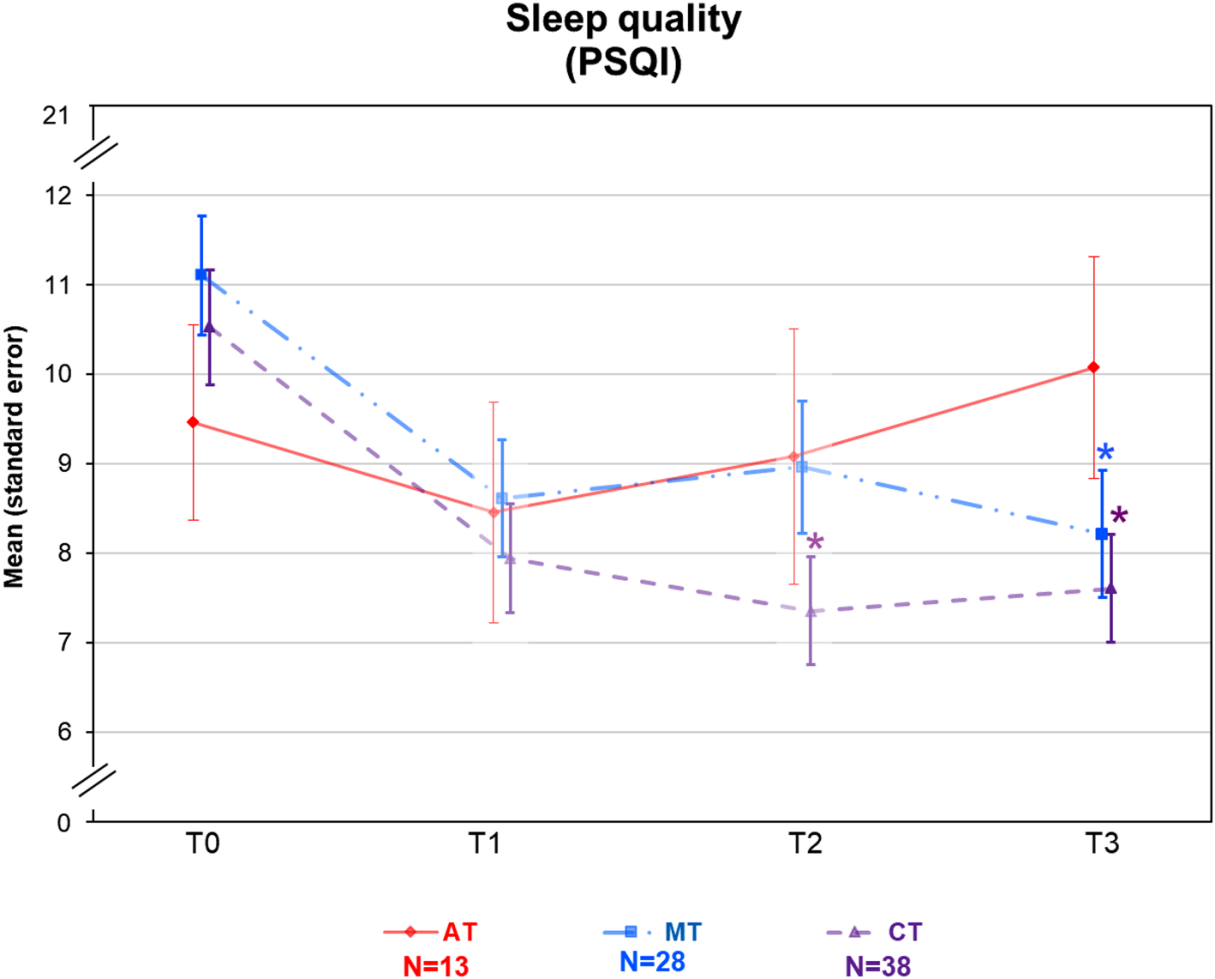
Course of the PSQI for the 3 study groups (aerobic training: red; multimodal therapy: blue; combination therapy: violet) over 4 years (n = 79), including after 10 weeks of intervention (T1) and 6 months later (T2) in frail color (n = 79). Colored asterisk indicates significant better sleep quality in MT and CT versus AT. *PSQI* Pittsburgh Sleep Quality Index, *AT* aerobic therapy, *MT* multimodal therapy, *CT* combination therapy.

Based on 278 breast cancer screened patients, 126 breast cancer patients with chronic CRF without metastases were included in the study from June 2011 to March 2013. Of those, 65 participants were randomized to 1 of the 3 treatment arms and 61 who refused randomization were allocated by preference (AT: 22 randomized/6 by preference; MT: 21/23; CT: 22/32)^23^. Out of the 105 participants starting the intervention and included in the ITT analysis, 84 (AT: 13; MT: 30; CT: 41) finished the 10-week intervention program (T1), 81 (AT: 13; MT: 28; CT: 40) answered six months later (T2) (reported in^23^). At the 4-year follow-up from May 2016 to February 2017 we invited all remaining participants who started the intervention and had neither withdrawn their consent to the study nor died. In total, n = 90 were asked to fill in the questionnaires. 79 responded (AT: 13; MT: 28; CT: 38), four participants had died (AT: 1; MT: 2; CT: 1) and the other patients did not answer, refused participation, or their address was no longer correct (for details see Fig. 1: flowchart). In this article we report the results of n = 79 study participants at baseline (T0) and at the 4-year follow-up (T3). To provide more information on aspects of the course and possible sustainability on the CFS-D and PSQI values, the results after 10 weeks of intervention (T1) and 6 months later (T2) in the 3 study groups for n = 79 are included in Fig. 2 and 3.

### Study group characteristics and long-term adherence

The sociodemographic and tumor data of the study groups of the remaining 79 patients were mostly comparable to the 105 who started the intervention. This was mostly true also between the three treatment arms with the following exceptions: combination therapy having received less chemotherapies (Table 1); multimodal therapy having a longer sleep latency (Table 3) and less HADS anxiety, less concentration disorders, less strain, and other disorders, and less expectation to improve professional life and higher values of achieve satisfaction; and aerobic training having less dyspnea at baseline (all *p* < 0.05). The mean age of these remaining patients ranged from 56.5 [standard deviation (SD) = 8.2] (CT) to 59.9 (SD = 9.5) (MT) years at baseline, with a mean time duration since first diagnosis of 1.9 (SD = 0.8) (CT) to 2.2 (SD = 0.8) years (MT) (Table 1).

**Table 1:** Socio-demographic characteristics at baseline (T0) for patients participating at the 4 years follow-up (T3), n = 79.

Treatment group	Aerobic therapy	Multimodal therapy	Combination therapy	*p*
Included	28	44	54	
Started intervention T0	20	34	51	
Completed T1	13	30	41	
Completed T2	13	28	40	
Completed T3	13	28	38	
**Marital status**				0.632
Single (%)	1 (7.7)	6 (21.4)	3 (7.9)	
Married (%)	9 (69.2)	13 (46.4)	22 (57.9)	
Divorced (%)	1 (7.7)	8 (28.6)	11 (29.0)	
Widowed (%)	2 (15.4)	1 (3.57)	2 (5.26)	
Children: yes (%)/ Children at home: yes (%)	10 (76.9)/ 4 (30.8)	21 (75.0)/ 7 (25.0)	30 (79.0)/ 8 (21.1)	0.835/ 0.819
**Employment**				0.279
Employed (%)	7 (53.9)	9 (32.1)	19 (50.0)	
Housewife (%)	0 (0.0)	3 (10.7)	1 (2.6)	
Unemployed (%)	0 (0.00)	1 (3.6)	4 (10.5)	
Pensioner (%)	3 (23.10)	10 (35.7)	9 (23.7)	
Sickness certificate (%)	2 (15.4)	3 (10.7)	3 (7.9)	
Other (%)	0 (0.0)	1 (3.6)	0 (0.0)	
Missing data (%)	1 (7.7)	1 (3.6)	2 (5.3)	
**Vocational education**				0.418
Apprenticeship (%)	5 (38.5)	12 (42.9)	14 (36.8)	
Technical College (%)	3 (23.1)	1 (3.6)	3 (7.9)	
University of Applied Sciences (%)	2 (15.4)	2 (7.1)	3 (7.9)	
University (%)	0 (0.0)	7 (25.0)	9 (23.7)	
No (%)	0 (0.0)	1 (3.6)	1 (2.6)	
Missing data (%)	3 (23.1)	5 (17.9)	8 (21.1)	
Age: Mean (SD)	58.2 (10.0)	59.9 (9.5)	56.5 (8.2)	0.274
Years since first diagnosis: Mean (SD)	2.0 (1.0)	2.2 (0.8)	1.9 (0.8)	0.353
Surgery: yes/%	13/100.0	28/100.0	38/100.0	
Chemotherapy: yes/%	8/61.5	20/71.4	15/39.5	0.032*
Years since chemotherapy: Mean (SD)	2.1 (0.7)	1.6 (0.6)	1.8 (0.7)	0.173
Radiotherapy: yes/%	11/84.6	23/82.1	29/76.3	0.756
Antihormonal therapy: yes/%	11/84.6	19/67.9	22/57.9	0.305
Mistletoe therapy: yes/%	4/30.8	6/21.4	10/26.3	0.801

Numbers and percentages relating to patients who completed T3 (n = 79),﻿ *: *p* < 0.05.

*SD* standard deviation.

At the 4-year follow-up, 2 participants in the multimodal therapy group and 1 in the combination therapy group were metastasized. 2 patients each in the multimodal therapy and combination therapy group had local recurrences, but none in the aerobic training group (shown in Table 2).

**Table 2:** Sociodemographic and disease-related sociodemographic data and therapy adherence after four years, n = 79.

Treatment group	Aerobic therapy		Multimodal therapy		Combination therapy		*p*
**Local cancer recurrence**							0.386
Yes	0	(0.00%)	2	(7.14%)	2	(0.00%)	
No	12	(92.31%)	26	(92.86%)	35	(92.11%)	
Unknown	1	(7.69%)	0	(0.00%)	0	(0.00%)	
**Metastases**							0.567
Yes	0	(0.00%)	2	(7.14%)	1	(2.63%)	
No	12	(92.31%)	25	(89.29%)	36	(94.74%)	
Unknown	1	(7.69%)	1	(3.57%)	1	(2.63%)	
**Therapies in the last 4 years #**							
Number of patients with surgery	1	(7.69%)	5	(17.86%)	5	(13.16%)	0.771
Surgery #	3		5		6		
**Indication according to patient information #**							
As a consequence of metastases	0		1		1		
As a consequence of tumor recurrence	0		2		1		
Indirectly related to breast cancer	0		1		2		
Other	0		1		0		
Not clearly assignable	3		0		2		
Chemotherapy	0	(0.00%)	1	(3.57%)	0	(0.00%)	0.435
Radiotherapy	0	(0.00%)	1	(3.57%)	2	(5.26%)	0.625
Antihormonal therapy	8	(61.54%)	16	(57.14%)	14	(36.84%)	0.199
Mistletoe therapy	4	(30.77%)	5	(17.86%)	12	(31.58%)	0.553
Missing data for mistletoe therapy	0	(0.00%)	2	(7.14%)	0	(0.00%)	
Missing data for therapies	0	(0.00%)	1	(3.57%)	0	(0.00%)	
**Hospitalization in the last 4 years**							
Mean value (SD)	4.0	(3.3)	1.9	(1.0)	2.4	(2.6)	0.359
	n = 6		n = 10		n = 11		
**Hospitalization in the last 4 years**							
No	2	(15.38%)	6	(21.43%)	12	(31.58%)	
1 Time	2	(15.38%)	4	(14.29%)	4	(10.53%)	
2 Times	0	(0.00%)	4	(14.29%)	6	(15.79%)	
3 Times	1	(7.69%)	1	(3.57%)	0	(0.00%)	
4 Times	2	(15.38%)	1	(3.57%)	0	(0.00%)	
5–10 Times	1	(7.69%)	0	(0.00%)	1	(2.63%)	
Missing data	5	(38.46%)	12	(42.86%)	15	(39.47%)	
**Sick days in the last 4 years**							
Mean value (SD)	40.4	(21.2)	52.9	(73.9)	92.2	(134.8)	0.647
	n = 5		n = 10		n = 11		
**Still employed**	4	(30.77%)	6	(21.43%)	11	(28.95%)	
**Days of incapacity to work in the last 4 years**							
0	0	(0.00%)	4	(14.29%)	7	(18.42%)	
1–14	1	(7.69%)	4	(14.29%)	2	(5.26%)	
15–29	0	(0.00%)	1	(3.57%)	4	(10.53%)	
30–59	2	(15.38%)	1	(3.57%)	1	(2.63%)	
60 and more	2	(15.38%)	4	(14.29%)	4	(10.53%)	
Missing data	8	(61.54%)	14	(50.00%)	20	(52.63%)	
**Therapies from study are still being carried out**							0.411
Yes	7	(53.85%)	17	(60.71%)	29	(76.32%)	
No	5	(38.46%)	10	(35.71%)	9	(23.68%)	
Missing data	1	(7.69%)	1	(3.57%)	0	(0.00%)	
**Therapies that are still being carried out after 4 years**							
Aerobic training	7	(53.85%)	9	(32.14%)	26	(68.42%)	
Psychoeducation	0	(0.00%)	6	(21.43%)	4	(10.53%)	
Sleep education	0	(0.00%)	6	(21.43%)	4	(10.53%)	
Eurythmy therapy	1	(7.69%)	6	(21.43%)	7	(18.42%)	
Painting therapy	1	(7.69%)	5	(17.86%)	6	(15.79%)	
**Frequency of the therapies that are carried out by the patients**							0.164
Daily	0	(0.00%)	1	(3.57%)	5	(13.16%)	
4–6 Times a week	2	(15.38%)	1	(3.57%)	3	(7.89%)	
1–3 Times a week	5	(38.46%)	13	(46.43%)	17	(44.74%)	
1–3 Times a month	1	(7.69%)	2	(7.14%)	5	(13.16%)	
Less frequently	0	(0.00%)	0	(0.00%)	0	(0.00%)	
Missing data	5	(38.46%)	11	(39.29%)	8	(21.05%)	

^#^ Multiple entries per patient possible.

*SD* standard deviation.

For the descriptive sociodemographic parameters, disease related data and oncological treatment of the 4-year follow-up we did not find any significant group differences (see Table 2).

The 4-year follow-up showed that 17 patients of the multimodal therapy group (60.7%), 29 of the combination therapy group (76.3%) and 7 of the aerobic training group (53.9%) still continued to carry out their treatments at least 1–3 times per month or even daily. The frequency of the different treatment groups and therapies in the three groups is presented in Table 2. The appraisal of the interventions was estimated in the multimodal therapy group by 10 patients as very effective (35.7%), by 11 (39.3%) as rather effective and by 6 (21.4%) as rather not or not effective. In the combination therapy group, 11 (28.9%) patients judged the therapies as very effective, 21 (55.3%) as rather effective and 6 (15.8%) as rather not or not effective. In the aerobic training group 1 patient (7.7%) estimated the treatment as very effective, 9 (69.2%) as rather effective and 3 (23.1%) as rather not effective (*p* = 0.569).

Regarding the effectiveness analysis, after 4 years, the primary outcome PC score of cancer fatigue (CFS-D)/sleep quality (PSQI)- still showed a sustainable, significant superiority of multimodal therapy (mean: 0.176, SD = 0.064) and combination therapy (mean: 0.178, SD = 0.062) compared to control aerobic training (mean: 0.206, SD = 0.066) with strong Cohen’s d standardized effect sizes (SES) of 1.04, respectively compared to aerobic training with SES = 0.37 (MT vs. AT: Δ_PC_ =  − 0.043, 95%-CI [− 0.082; − 0.004], *p* = 0.033; CT vs. AT: Δ_PC_ =  − 0.040, 95%-CI [− 0.078; − 0.003], *p* = 0.035). The results of the baseline (T0), after the intervention (T1) and 6 months later (T2) are presented in a former article^23^ indicating, along with this data, the explorative sustainable superiority of multimodal therapy versus aerobic training at T1, T2 and T3 and of combination therapy versus aerobic training at T2 and T3. In the randomized patients we found a significant superiority for multimodal and combination treatment compared to aerobic training after 4 years (see Table 4).

The improvement of fatigue in the CFS-D total-score was still at a comparable level in this 4-year follow-up to the end of the intervention after 10 weeks (T1) and 6 months later (T2) in the multimodal therapy and combination therapy group. This result underlines sustainability with a mean difference in the multimodal therapy group of − 8.3 (SD = 7.3; SES = 0.96) and in combination therapy of − 8.2 (SD = 9.3; SES = 0.95) 4 years later and was still significantly improved compared to baseline even if not superior compared to the aerobic training group (− 7.4, SD = 7.5; SES = 0.78) (Table 3)^23^. This is related to the improvement of aerobic training from the 6-month follow-up to T3 after 4 years. In the CFS-D subscales physical, cognitive and affective fatigue we exploratively detected significant intra-group improvements in multimodal therapy and combination therapy, and in cognitive fatigue even in aerobic training, but we found no significant differences after 4 years between the groups for the whole sample and for randomized participants (Tables 3, 4, Fig. 2).

**Table 3:** CFS-D and PSQI baseline data and T3–T0 differences (with standard deviation), n = 79.

	Aerobic therapy	Multimodal therapy	*p* (MT vs. AT)	Combination therapy	*p* (baseline^#^) *p* (CT vs. AT^†^)
**CFS-D total score**					
Baseline T3–T0.01	35.3 (8.5) − 7.3 (7.5)*	33.5 (7.3) − 8.3 (7.3)*	0.245	35.1 (7.7) − 8.2 (9.3)*	0.311^#^ 0.378^**†**^
**PSQI total score**					
Baseline T3–T0.01	9.5 (3.95) 0.6 (2.7)	11.1 (3.5) − 2.9 (4.0)*	**0.020**	10.5 (4.0) − 2.9 (3.9)*	0.484^#^ **0.007**^**†**^
**CFS-D physical**					
Baseline T3–T0.01	16.2 (2.3) − 2.5 (3.6)	15.7 (2.8) − 3.1 (3.6)*	0.176	15.5 (3.6) − 3.5 (4.5)*	0.889^#^ 0.127^**†**^
**CFS-D cognitive**					
Baseline T3–T0.01	11.5 (4.5) − 3.3 (2.5)*	10.7 (4.2) − 3.0 (3.8)*	0.674	12.05 (3.0) − 2.8 (3.1)*	0.257^#^ 0.788^**†**^
**CFS-D affective**					
Baseline T3–T0.01	7.6 (2.2) − 1.6 (2.2)	7.1 (2.2) − 2.1 (2.3)*	0.138	7.5 (2.3) − 2.0 (3.1)*	0.557^#^ 0.322^**†**^
**PSQI subjective sleep quality**					
Baseline T3–T0.01	1.58 (0.51) 0.17 (0.58)	1.89 (0.63) -0.57 (0.63)*	**0.007**	1.78 (0.72) -0.47 (0.74)*	0.394^#^ **0.007**^**†**^
**PSQI sleep latency**					
Baseline T3–T0.01	1.46 (1.05) 0.46 (0.88)	**2.19 (0.88)** − 0.88 (0.86)*	**0.005**	1.53 (1.08) − 0.25 (1.00)*	**0.028**^#^ **0.024**^**†**^
**PSQI sleep duration**					
Baseline T3–T0.01	1.23 (1.01) 0.15 (0.69)	1.37 (1.11) − 0.22 (1.28)	0.385	1.34 (1.15) − 0.45 (1.31)*	0.933^#^ 0.127^**†**^
**PSQI sleep efficiency**					
Baseline T3–T0.01	1.23 (1.09) 0.15 (0.90)	1.85 (1.23) − 0.52 (1.45)	0.421	1.55 (1.11) − 0.66 (1.12)*	0.262^#^ 0.085^**†**^
**PSQI sleep disturbances**					
Baseline T3–T0.01	1.69 (0.75) 0.08 (0.64)	1.89 (0.57) − 0.43 (0.57)*	**0.031**	1.74 (0.55) − 0.26 (0.60)*	0.473^#^ 0.076^**†**^
**PSQI taking hypnotics**					
Baseline T3–T0.01	0.38 (0.96) 0.00 (1.15)	0.18 (0.61) 0.07 (0.47)	0.846	0.47 (1.06) − 0.13 (0.58)	0.629^#^ 0.745^**†**^
**PSQI daytime sleepiness**					
Baseline T3–T0.01	2.00 (0.85) − 0.45 (0.82)	1.81 (0.85) − 0.38 (0.80)*	0.414	2.11 (0.74) − 0.65 (0.79)*	0.381^#^ 0.220^**†**^

^#^Differences between the 3 groups at baseline are tested with the van Elteren test (see baseline row, last column).

*Significant changes from Baseline to T3; significant differences between MT and AT (see T3–T0.01 row, third column) or CT and AT (see T3-T0.01 row, last column) are presented in bold. The *p* value CT versus AT is specified with^†^.

*AT* Aerobic therapy, *MT* multimodal therapy, *CT* combination therapy, *CFS-D* cancer fatigue scale, German version, *PSQI* Pittsburgh Sleep Quality Index.

**Table 4:** Standardized effect sizes (Cohens d) of changes between baseline (T0) and four years later (T3) regarding CFS-D and PSQI for randomized patients, n = 39.

	Aerobic therapy	Multimodal therapy	Combination therapy
Composite score CFS-D/PSQI	0.36	1.64*	1.11*
CFS-D total score	0.75	1.51	0.72
PSQI total score	−0.09	1.15*	0.97*

*p < 0.05 for MT versus AT and CT versus AT.

*CFS-D* cancer fatigue scale German version, *PSQI* Pittsburgh Sleep Quality Index.

Regarding the PSQI total score, the sleep quality was still superior in multimodal therapy (mean difference =  − 2.9, SD = 4.0; SES = 0.79) and combination therapy (mean difference =  − 2.9, SD = 3.9; SES = 0.76) compared to aerobic training (mean difference = 0.6, SD = 2.7; SES = −0.15) and measured a sustainably improved sleep quality with significant improvement compared to baseline in multimodal therapy (*p* < 0.0001) and combination therapy (*p* < 0.0001), but not in aerobic training (Table 3, Fig. 3)^23^. This was also true for the randomized patient groups (Table 4). Patients treated in the multimodal therapy and combination therapy group improved compared to baseline and achieved exploratively better sleep quality and shorter sleep latency and in multimodal therapy even fewer sleep disturbances compared to aerobic training (all *p* < 0.05) (Table 3). Furthermore, patients in the combination therapy group improved compared to baseline regarding sleep duration, sleep efficiency and disturbances, and together with multimodal therapy regarding daytime sleepiness without achieving superiority to aerobic training (shown in Table 3).

## Discussion

Breast cancer patients with chronic CRF treated over 10 weeks with a multimodal intervention program including psychoeducation, sleep education, eurythmy and painting therapy and a combined multimodal therapy with aerobic training showed strong standardized effect-sizes and a sustainable superiority in the primary outcome fatigue/sleep quality compared to trainer-guided standard aerobic training even after 4 years of follow-up. This applied to the entire study group and also to the randomized subgroup.

As published previously, even if the combination therapy failed to show confirmative superiority after 10 weeks of intervention (T1), in an explorative analysis compared to aerobic training the multimodal therapy was already superior to aerobic training at the end of the intervention (T1) and, together with combination therapy, 6 months later (T2)^23^. This superiority remained sustainable over 4 years for both groups, multimodal therapy and combination therapy. However, this long-term superiority was particularly related to the improvements in the PSQI-global sleep quality, including improved sleep onset latency, sleep quality in multimodal therapy and combination therapy and in multimodal therapy fewer sleep disturbances and the lack of improved sleep quality in aerobic training. Regarding the CFS-D, the multimodal treatment was superior to aerobic treatment after six months^23^ but not after 4 years. This is true despite the fact that in both experimental groups remarkably sustainable long-term improvements with strong standardized effect-sizes were observed after 4 years regarding global fatigue, physical and affective fatigue. More than half of the patients of the multimodal therapy group and 38% of the combination therapy group were below the CFS-D inclusion threshold of 24. However, in the aerobic training group the fatigue level decreased considerably between 6 months^23^ and 4 years later, hence one third of these patients achieved a CFS-D level below their inclusion level.

Precipitating factors for CRF are cancer treatments such as chemo- or radiotherapy, surgery or anti-hormonal therapies and tumor burden^40,41^. Some hypotheses are discussed to be involved in the pathophysiology of CRF such as pro-inflammatory, hypothalamic–pituitary–adrenal axis disruption, circadian rhythm disruption, serotonin and vagal afferent nerve function as central and peripheral mechanisms^42^. Particularly, alterations in the sleep/wake rhythm and an altered neuroendocrine system with flattened cortisol curve, reduced glucocorticoid sensitivity and responsiveness, as well as autonomic dysregulation have been discussed as perpetuating factors for chronic inflammation and an associated increase of cytokine release and CRF^41,43^. This model may help to explain the complex neuro-endo-immunological alteration being associated with CRF, including fatigue, sleep and cognitive dysfunction and distress^41,43^. Single interventions for the treatment of CRF have only a limited effectiveness with low to moderate standardized effect sizes^17^, with cognitive fatigue being particularly difficult to treat, e.g., using endurance or resistance exercise^44^. In breast cancer patients who received adjuvant chemotherapy, performed worse on different neurocognitive functions and tests, and cognitive complaints are subjectively present in 38% (word finding) to 52% (problems remembering) of all patients, even after 20 years^16^.

With our multimodal treatment we wanted to address this system of complex dysregulation at different levels. Even after 4 years, patients in the 3 groups improved without group differences. With the help of sleep education^26^, sleep restriction^27^ and stimulus control we wanted to support normalizing the sleep quality by reducing time-in-bed, sleep onset latency and improving sleep continuation and restorative sleep, and hence promote the rest/activity resynchronization and sleep quality^22,45^. Even if the impact of the sleep intervention remains unclear, these sleep parameters were improved in the multimodal therapy group compared to the AT group. This is coherent with another RCT study which evidenced a significant superiority of CBT sleep intervention in breast cancer patients compared to a healthy eating breast cancer control group regarding PSQI sleep quality, but not Pipers Fatigue Scale directly after the intervention and one year later, even if the fatigue level was still improved at that time^19^. However, in our study the improved sleep quality was sustainable over 4 years.

With the help of psychoeducation, it was intended to improve patients’ knowledge of CRF and self-management capacity regarding the high distress level^46^. Even if, as published in another article, we did not show an improvement in the anxiety and distress in the experimental groups after the intervention^47^ and standardized effect sizes of CBT on fatigue are estimated as minor according to meta-analyses^17,18^, the patients in the multimodal therapy and combination therapy groups improved in self-regulation and internal coherence^47^. Additional qualitative data suggests that patients felt more competent about handling daily challenges of life^47^.

For physical exercise an improvement of cardiopulmonary^48^, physical performance^32^ and, based on a large number of studies, a minor standardized effect size on fatigue^17,49^ is evidenced, and, together with cognitive behavioral therapies, it may have limiting influence on inflammation markers related to CRF^43,50^.

There is sufficient evidence that mindfulness-based therapies can improve the relaxation response^51^, fatigue^52^, sleep quality and distress^53,54^ and might reduce sympathetic nervous system signaling^50^ and pro-inflammatory cytokines such as TNF-alpha^50^. However, evidence is still missing on the extent to which eurythmy therapy and painting therapy as mindfulness-oriented treatments as stand-alone interventions can reduce fatigue, sleep quality and distress in breast cancer patients with CRF. However, studies on eurythmy therapy have shown a reduction of stress and improvement of quality of life criteria such as emotional functioning and mental health^55^. Studies on painting therapy have shown reduced depression scores in patients undergoing chemotherapy^21^. However, it remains unclear to which degree these interventions might have contributed to the long-term improvement of sleep quality and fatigue within our multimodal and combination treatment. The German Society of Cancer in its current guideline for complementary oncology has evaluated the ten week and six month data and recommended that this multimodal therapy “can be considered in breast cancer patients” to treat fatigue and difficulties falling asleep and staying asleep^56^. The strong standardized effect-size of the multimodal and combination treatment even after four years implies sustainable higher effect-sizes than the mostly low to moderate short-medium term standardized effect-sizes of single treatments^17^. This is encouraging to provide a multimodal treatment in the context of integrative oncology or rehabilitation medicine.

One of the strengths of our study is the long-term follow-up, along with the three study time-points outlining the sustainability of improved fatigue/sleep quality and global sleep quality in the multimodal and combined treatment group^23^. Other strengths are the adherence of more than half of the patients in all groups over 4 years, continuing with one or more exercises learnt at the beginning of the study at least 1–3 times a week, and the high response rate at the 4-year follow-up. This indicates a profound sustainable change in patients’ self-management. In another article the improved self-regulation, e.g., the behavior ‘to reach goals and achieve satisfaction’ and, delayed after 6 months, the internal coherence, including the inner coherence and resilience and thermo-coherence subscales^47^ and the improved rest/activity and autonomic regulation in the multimodal treatment group^57^ reflect the improved self-management and circadian regulation.

However, our study also has a number of limitations: we have to emphasize the relatively small number of study participants, especially in the aerobic training group. In addition, the internal validity of the study is reduced by the inclusion of patients who have been assigned to a therapy group based on their preference which may introduce an unidentified allocation bias, even though this increased the external validity. The different time durations of the trainer-guided therapy sessions in the 3 therapy arms present another weakness. While the combination therapy group received 1810 min and the multimodal therapy group 1,450 min, the trainer-guided treatment sessions of the aerobic training group were 360 minutes^23^. Even if the aerobic training group had the longest home-based exercise and training times with 223 min/week, there are still differences in trainer contact times^23^. Finally, the drop-out rate of about 20% after the start of the intervention is high, especially in the aerobic training group. However, our data is within the range of other published drop-out rates for endurance therapy studies^58^. Finally, the generalizability of the results is limited to patients with breast cancer with (chronic) CRF and without metastases.

In conclusion, to confirm the long-term explorative superiority of the multimodal treatment concept on the combined outcome sleep quality and fatigue, global sleep quality and the sustainable improvement of CRF, a confirmative randomized controlled trial is highly warranted. There, however, the therapy dosage in the combination therapy group should be adapted thoroughly for movement therapies as we discussed in a former article^23^ and the health-economic costs of such multidisciplinary approaches must be calculated. In case of confirmation of superiority of the multimodal therapy concept in a larger RCT trial it has the potential to reduce long-term fatigue. Based on the actual available evidence, it is already intriguing that breast cancer patients with CRF now have the choice of being treated with mindfulness-oriented approaches instead of aerobic training alone, with comparable standardized effect sizes^59^ or e.g. with the multimodal therapy with even higher standardized effect sizes and long-term effects on sleep quality and fatigue.

## Data Availability

Any request for data and materials should be made in writing to the corresponding author, and these will be considered.
